# Lenvatinib for the treatment of hepatocellular carcinoma—a real-world multicenter Australian cohort study

**DOI:** 10.1007/s12072-022-10398-5

**Published:** 2022-08-25

**Authors:** Kurvi Patwala, David Stephen Prince, Yael Celermajer, Waafiqa Alam, Eldho Paul, Simone Irene Strasser, Geoffrey William McCaughan, Paul Gow, Siddharth Sood, Elise Murphy, Stuart Roberts, Elliot Freeman, Elizabeth Stratton, Scott Anthony Davison, Miriam Tania Levy, McCawley Clark-Dickson, Vi Nguyen, Sally Bell, Amanda Nicoll, Ashley Bloom, Alice Unah Lee, Marno Ryan, Jessica Howell, Zina Valaydon, Alexandra Mack, Ken Liu, Anouk Dev

**Affiliations:** 1 Department of Gastroenterology, Monash Health, 246 Clayton Road, Clayton, VIC 3168 Australia; 2 AW Morrow Gastroenterology and Liver Centre, Royal Prince Alfred Hospital, 50 Missenden Road, Camperdown, NSW 2050 Australia; 3 Department of Gastroenterology, Liverpool Hospital, 75 Elizabeth Street, Liverpool, NSW 2170 Australia; 4 Department of Medicine, Monash University, Wellington Road, Clayton, VIC 3800 Australia; 5 Department of Gastroenterology and Liver Transplant, Austin Health, 145 Studley Road, Heidelberg, VIC 3084 Australia; 6 Department of Gastroenterology, Royal Melbourne Hospital, 300 Grattan Street, Parkville, VIC 3050 Australia; 7 Department of Gastroenterology, Alfred Health, 55 Commercial Road, Melbourne, VIC 3004 Australia; 8 Department of Gastroenterology, Royal North Shore Hospital, Reserve Road, St Leonard’s, NSW 2065 Australia; 9 Department of Gastroenterology, Eastern Health, 8 Arnold Street, Box Hill, VIC 3128 Australia; 10 Department of Gastroenterology, Concord Repatriation General Hospital, Hospital Road, Concord, NSW 2139 Australia; 11 Department of Gastroenterology, St Vincent’s Hospital, 41 Victoria Parade, Fitzroy, VIC 3065 Australia; 12 Department of Medicine, University of Melbourne, Parkville, VIC 3010 Australia; 13 Disease Elimination Program, Burnet Institute, 85 Commercial Road, Melbourne, VIC 3004 Australia; 14 Department of Epidemiology and Preventative Medicine, Monash University, Wellington Road, Clayton, VIC 3800 Australia; 15 Department of Gastroenterology, Western Health, 160 Gordon Street, Footscray, VIC 3011 Australia

**Keywords:** Liver malignancy, Adverse effects, Diarrhoea, Hypertension, Multikinase inhibitor, Chronic liver disease, Systemic therapy, Oral therapy, Australia, Cohort

## Abstract

**Introduction:**

Hepatocellular carcinoma (HCC) is a serious complication of chronic liver disease. Lenvatinib is an oral multikinase inhibitor registered to treat advanced HCC. This study evaluates the real-world experience with lenvatinib in Australia.

**Methods:**

We conducted a retrospective cohort study of patients treated with lenvatinib for advanced HCC between July 2018 and November 2020 at 11 Australian tertiary care hospitals. Baseline demographic data, tumor characteristics, lenvatinib dosing, adverse events (AEs) and clinical outcomes were collected. Overall survival (OS) was the primary outcome. Progression free survival (PFS) and AEs were secondary outcomes.

**Results:**

A total of 155 patients were included and were predominantly male (90.7%) with a median age of 65 years (interquartile range [IQR]: 59–75). The main causes of chronic liver disease were hepatitis C infection (40.0%) and alcohol-related liver disease (34.2). Median OS and PFS were 7.7 (95% confidence interval [CI]: 5.8–14.0) and 5.3 months (95% CI: 2.8–9.2) respectively. Multivariate predictors of mortality were the need for dose reduction due to AEs (Hazard ratio [HR] 0.41, *p* < 0.01), new or worsening hypertension (HR 0.42, *p* < 0.01), diarrhoea (HR 0.47, *p* = 0.04) and more advanced BCLC stage (HR 2.50, *p* = 0.04). Multivariable predictors of disease progression were higher Child–Pugh score (HR 1.25, *p* = 0.04), the need for a dose reduction (HR 0.45, *p* < 0.01) and age (HR 0.96, *p* < 0.001). AEs occurred in 83.9% of patients with most being mild (71.6%).

**Conclusions:**

Lenvatinib remains safe and effective in real-world use. Treatment emergent diarrhoea and hypertension, and the need for dose reduction appear to predict better OS.

**Supplementary Information:**

The online version contains supplementary material available at 10.1007/s12072-022-10398-5.

## Introduction

Hepatocellular carcinoma (HCC) is a serious complication of chronic liver disease with a worldwide incidence of 10.1 cases per 100,000 person-years [1]. It is the most common type of liver cancer, and the second leading cause of malignancy related mortality globally [2, 3]. In Australia, HCC is the 11th most common cancer in males and 20th in women, but it is one of the most rapidly rising causes of cancer death with the incidence increasing more than four fold in the last 30 years [4].

The treatment for HCC is influenced by a range of factors as outlined by the Barcelona Clinic Liver Cancer (BCLC) staging system. In patients with advanced-stage disease, compensated liver function and preserved functional status, systemic therapies are the recommended first line treatment [5–7]. Lenvatinib is a multi-target oral multi-kinase inhibitor with activity against multiple carcinogenesis pathways. It was widely approved as first-line systemic therapy for advanced HCC after it was shown in a large phase III clinical trial to be non-inferior to sorafenib in terms of overall survival (OS) with improved progression free survival (PFS) [8]. Following its inclusion in the Australian pharmaceutical benefits scheme (PBS) in March 2019, lenvatinib overtook sorafenib in June 2019 as the most commonly prescribed agent for advanced HCC, peaking at approximately 75% of all prescriptions for HCC in mid-2020 [9].

Despite the emergence of combination infusional therapy with atezolizumab and bevacizumab, lenvatinib monotherapy is likely to have an ongoing place in the management of HCC in patients for whom atezolizumab and/or bevacizumab are contraindicated or in those without access to infusional therapy [10]. Moreover, several phase III trials of immunotherapy combinations are ongoing, including a study of an anti-PD1 inhibitor in combination with lenvatinib [11]. In some countries, particularly in the Asia–Pacific region which has the highest global burden of HCC, lenvatinib may also be more affordable than immunotherapy. Thus, it is important to have local real-world data evaluating its use, effectiveness and safety.

To date, retrospective studies evaluating real-world experience with lenvatinib in advanced HCC have been limited to descriptive studies or from regions with a homogenous ethnic population [12–18].We aim to evaluate the characteristics and safety of lenvatinib treatment for advanced HCC in a multi-ethnic population in eleven Australian tertiary referral centres.

## Materials and methods

This was a retrospective, multi-centre, cross-sectional study of patients who received lenvatinib therapy for advanced stage HCC from July 2018 to November 2020. Eligible patients were recruited from pharmacy and HCC databases and had a confirmed HCC diagnosed in accordance with the American Association for Study of Liver Diseases clinical practice guidelines [19]. Patients were included if they had received at least one dose of lenvatinib and had been followed up post commencement of therapy. Patients who had previously undergone liver transplant and had HCC recurrence (*n* = 5) or patients with fibrolamellar HCC (*n* = 2) were excluded. Baseline demographic data, tumor characteristics, lenvatinib dosing, adverse events and clinical outcomes were collected from medical records, pharmacy records and centralised databases.

Patients were recommended to commence the appropriate weight-based dose of lenvatinib (12 mg/day for patients greater than or equal to 60 kg body weight and 8 mg/day for those less than 60 kg body weight). In practice this may have varied based on clinician discretion. The starting and maximum dose for each patient and the need for any changes in dose were recorded.

Progression was assessed radiologically using cross sectional imaging in accordance with mRECIST 1.1. AEs were noted and graded according to the Common Terminology Criteria for Adverse Events (CTCAE) v5.0 [20].

### Statistical analysis

The primary outcome of interest was overall survival (OS) which was determined from commencement of lenvatinib therapy to death from any cause. Patients who were lost to follow up were censored at time of their last healthcare interaction. Additionally, progression-free survival (PFS) was measured from commencement of therapy to date of radiological progression or to death by any cause.

Continuous variables were summarised using mean ± standard deviation (SD) or median (interquartile range [IQR]) as appropriate. Categorical variables were expressed as counts and proportions. Comparisons between groups were performed using Chi square or Fisher’s exact test for categorical variables and Student’s *t*-test or Mann–Whitney *U* test as appropriate for continuous variables. The Kaplan–Meier method was used to analyse survival as a function of time and the curves were compared using the log-rank test. Univariate and multivariate analysis for OS and PFS were performed using Cox proportional hazards regression to estimate hazard ratios (HR) and 95% confidence intervals (95% CI), relating variables to all-cause mortality. Variables with *p* < 0.05 on univariate analysis or those deemed to be clinically important were entered into a hierarchical regression model to identify factors independently associated with all-cause mortality. A *p* value < 0.05 was considered to be statistically significant. Analyses were performed with Statistical Analysis System SAS version 9.4 (SAS institute, Cary, USA) [21].

## Results

### Patient characteristics

A total of 155 patients across 11 sites were included. Patients were predominately male (90.7%) and Caucasian (60.6%) with a median age of 65 years (IQR: 59–75). Most patients had compensated Child–Pugh A (CP A) cirrhosis (78.8%). The main causes of chronic liver disease were hepatitis C infection (HCV) (40.0%), alcohol-related liver disease (34.2%), non-alcoholic fatty liver disease (NAFLD) (25.8%) and hepatitis B infection (HBV) (19.8%). Baseline patient characteristics are summarised in Table 1.

**Table 1:** Patient characteristics at time of commencement of lenvatinib

	*n* = 155 (%)
Age, years (IQR)	65 (59–75)
Male gender	141 (90.7)
Ethnicity	
Caucasian	94 (60.6)
Asian	34 (21.9)
Middle Eastern	7 (4.5)
Mediterranean	7 (4.5)
Subcontinental	5 (3.2)
African	2 (1.3)
Other	6 (3.9)
Aetiology^a^	
HCV	62 (40.0)
Alcohol	53 (34.2)
NAFLD	40 (25.8)
HBV	32 (20.6)
Other	14 (9.0)
Cirrhosis	139 (89.7)
Child–Pugh class and score	*n* = 137
Child Pugh A	108 (78.8)
CPA5	49 (35.8)
CPA6	59 (43.1)
Child Pugh B	27 (19.7)
CPB7	21 (15.3)
CPB8	6 (4.4)
CPB9	0 (0)
Child Pugh C	2 (1.5)
CPC10	2 (1.5)
MELD score, median (IQR)	8 (7–11)
ECOG status	
0	74 (47.7)
1	38 (24.5)
2	10 (6.5)
Unknown	33 (21.3)
Baseline blood tests	
AFP (ng/mL), median (IQR)	34.1 (5–633.6)
Creatinine (μmol/L), median (IQR)	76 (65–93)
Bilirubin (μmol/L) median, (IQR)	15 (10–23)
Albumin (g/L) median, (IQR)	35 (32–37)
INR	1.1 (1–1.2)
Ascites	
Absent	130 (83.9)
Mild	14 (9.0)
Moderate	6 (3.9)
Unknown	5 (3.2)
Encephalopathy	
Absent	147 (94.8)
Grade 1/2	3 (1.9)
Grade 3/4	0 (0)
Unknown	5 (3.2)

*ECOG* Eastern Cooperative Oncology Group; *HBV* hepatitis B virus; *HCV* hepatitis C virus; *INR* international normalised ratio; *NAFLD* non-alcoholic fatty liver disease; *MELD* model for end-stage liver disease AFP

^a^46 patients had two different aetiologies of liver disease (29.7%)

### Tumor characteristics

Almost all patients had BCLC stage C (69.7%) or BCLC stage B (27.7%) disease. Tumor thrombus was present in forty-four patients (28.4%). A total of 98 patients (63.2%) had received prior treatment for HCC and 54 patients (34.8%) were treatment naïve. Of those with treatment experience, 61 patients (60.4%) had previously received one treatment modality, 30 patients (29.7%) had received two different treatment modalities and seven patients (6.9%) had received three different treatment modalities. The majority of patients (80.6%) treated with lenvatinib had multiple liver lesions. Patients with only a single liver lesion (*n* = 30, 19.4%) had extra-hepatic metastatic disease (*n* = 15), portal vein invasion (*n* = 8), previous treatment experience leading to treatment stage migration (*n* = 4) or a very large lesion not suitable for locoregional therapy (*n* = 3). Tumor characteristics are summarised in Table 2.

**Table 2:** Tumor characteristics at time of commencement of lenvatinib

Number of intra-hepatic lesions	
One	30 (19.4)
Two	25 (16.1)
Three	16 (10.3)
Multifocal	78 (40.3)
Extra-hepatic at diagnosis	5 (3.2)
Unknown	1 (0.6)
Extra-hepatic metastases	60 (38.7)
Median size of largest lesion (mm) (IQR)	44 (23.25–90)
Portal vein thrombosis	
No thrombosis	96 (61.9)
Bland thrombus Vp1/Vp2	3 (1.9)
Bland thrombus Vp3/Vp4	10 (6.5)
Tumor thrombus Vp1/Vp2	10 (6.5)
Tumor thrombus Vp3/Vp4	34 (21.9)
Unknown	2 (1.3)
BCLC stage	
Stage A	2 (1.3)
Stage B	43 (27.7)
Stage C	108 (69.7)
Unknown	2 (1.3)
Treatment history	
Treatment experienced	98 (63.2%)
Treatment naïve	54 (34.8)
Unknown	3 (1.9)
Prior treatment details	
Locoregional therapy^a^	75 (48.4)
Selective internal radiation therapy	12 (7.7)
Resection	24 (15.5)
SBRT to liver lesions	4 (2.6)
Sorafenib	27 (17.4)

*AFP* alpha-foetoprotein; *BCLC* Barcelona Clinic Liver Cancer; *RFA* radiofrequency ablation; *MWA* microwave ablation; *TACE* transarterial chemoembolization; *SBRT* stereotactic radiotherapy to liver lesions

^a^Locoregional therapy defined as previous trans-arterial (chemo)embolisation or ablation procedures

### Lenvatinib dosing and duration of use

Patients were prescribed a starting dose of either 12 mg (41.3%), 8 mg (31.6%) or 4 mg (24.5%) daily. The maximum appropriate weight-based daily dose was reached in 65.2% of patients. A temporary interruption to therapy occurred in 36 patients (23.2%) due to intolerance. A total of 67 patients (43.2%) required dose reduction due to development of AEs. The median duration of therapy overall was 5.0 months (IQR 2.3–8.4 months). At time of analysis, therapy had been permanently ceased in 115 patients (74.2%) while 40 remained on therapy (25.8%). A total of 58 patients stopped therapy due to intolerance (50.4%), 44 stopped due to disease progression (38.3%) and 10 patients died while on therapy (8.7%).

### Overall survival

Patients were followed for a median period of 9.4 months (IQR 5.8–14.4) and during this period there were 83 deaths (53.5%). The median OS was 7.7 months (IQR 4.8–11.6 months) and the median PFS was 5.3 months (IQR 2.8–9.2 months).

### Predictors of overall survival

**Fig. 1:**
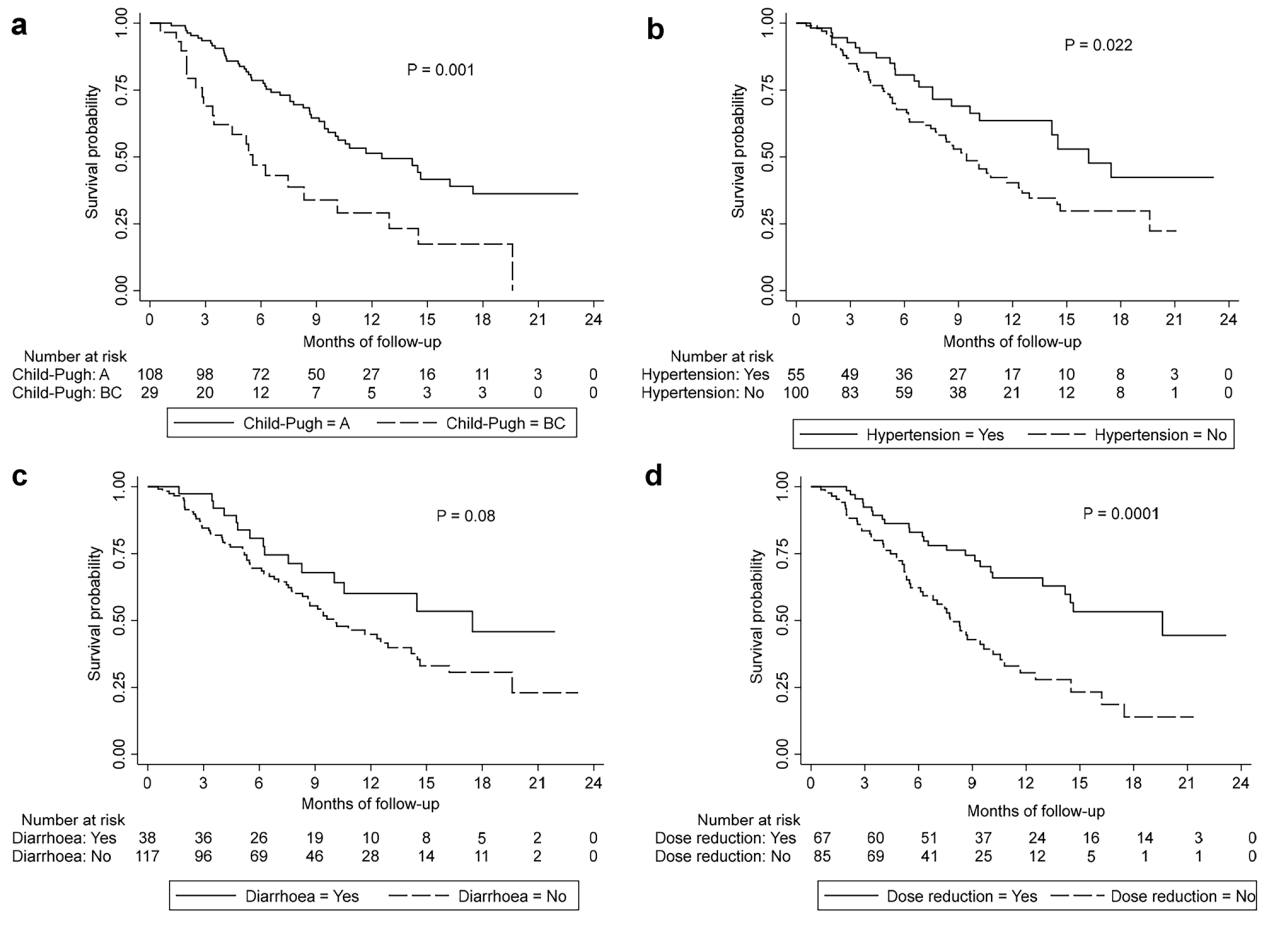
Kaplan–Meier curves for overall survival stratified by Child Pugh status (**a**) development of new or worsening hypertension (**b**) development of diarrhoea (**c**) and the need for a dose reduction due to AE (**d**)

Kaplan–Meier survival analysis (Fig. 1) revealed the development of new or worsening hypertension was associated with improved OS compared to those who did not develop hypertension (median OS 16.2 vs 9.4 months (*p* = 0.02)). Patients with treatment emergent diarrhoea also had an improved median OS compared to those without an altered bowel habit (17.5 vs 10.1 months (*p* = 0.08)). Additionally, a dose reduction due to the development of AEs was associated with improved survival compared to those who maintained stable dosing (19.6 vs 7.8 months respectively (*p* < 0.01)). Conversely decompensated liver disease was associated with worse OS when CP B/C patients were compared with CP A patients (median OS 5.6 vs 12.5 months (*p* < 0.01)).

Predictors of worse OS on univariate analysis were baseline CP score (HR 3.00, 95% CI 1.34–2.01, *p* < 0.01), MELD score (HR 1.10, 95% CI 1.03–1.17, *p* < 0.01), BCLC stage (HR 1.70, 95% CI 1.01–2.86, *p* = 0.04), ECOG score (HR 1.58, 95% CI 1.06–2.36, *p* = 0.02), presence of ascites at baseline (HR 1.86, 95% CI 1.01–3.43, *p* = 0.04), increased bilirubin (HR 1.02, 95% CI 1.01–1.04, *p* < 0.01) or presence of tumor thrombus (HR 1.89, 95% CI 1.19–3.01, *p* < 0.01). Predictors of improved OS were lenvatinib dose reduction (HR 0.38, 95% CI 0.23–0.63, *p* < 0.01) or withholding therapy (HR 0.44, 95% CI 0.24–0.82, *p* < 0.01), a higher baseline albumin (HR 0.89, 95% CI 0.85–0.93, *p* < 0.01) and the development of hypertension (HR 0.56, 95% CI 0.34–0.94, *p* = 0.02). There was a trend towards improved OS with the development of diarrhoea (HR 0.61, 95% CI 0.34–1.08, *p* = 0.08). There was no significant co-linearity between factors. Racial background, etiology of liver disease, previous exposure to any HCC therapy including sorafenib or reaching maximum dose of therapy did not impact overall survival.

On multivariate analysis, BCLC stage (HR 2.50 95% CI 1.40–4.45, *p* < 0.01), baseline albumin (HR 0.89, 95% CI 0.86–0.93, *p* < 0.01), the development of hypertension (HR 0.42, 95% CI 0.24–0.73, *p* < 0.01) or diarrhoea (HR 0.47, 95% CI 0.25–0.88, *p* = 0.01) and dose reduction (HR 0.41, 95% CI 0.24–0.69, *p* < 0.01) remained independent predictors for OS (Table 3).

**Table 3:** Univariate and multivariate analysis of factors predictive of mortality

	Univariate analysis			Multivariate analysis		
	HR	95% CI	*p*	aHR	95% CI	*P*
Dose reduction (yes vs. no)	0.38	0.23–0.63	< 0.01	0.41	0.24–0.69	< 0.01
Baseline albumin (per g/L increase)	0.89	0.85–0.93	< 0.01	0.89	0.86–0.93	< 0.01
Development of HTN as an adverse event (yes vs. no)	0.56	0.34–0.94	0.02	0.42	0.24–0.73	< 0.01
Development of diarrhoea as an adverse event (yes vs. no)	0.61	0.34–1.08	0.08	0.47	0.25–0.88	0.01
BCLC stage (per stage increase)	1.70	1.01–2.86	0.04	2.50	1.40–4.45	< 0.01
Treatment withheld (yes vs. no)	0.44	0.24–0.82	< 0.01			NS
Baseline MELD score (per point increase)	1.10	1.03–1.17	< 0.01			NS
Tumor thrombus (yes vs. no)	1.89	1.19–3.01	< 0.01			NS
Baseline CP score (per one-point increase)	3.00	1.34–2.01	< 0.01			NS
Baseline bilirubin (per μmol/L increase)	1.02	1.01–1.04	< 0.01			NS
ECOG score (each one-point increase)	1.58	1.06–2.36	0.02			NS
Ascites (yes vs. no)	1.86	1.01–3.43	0.04			NS

*HTN* hypertension; *BCLC *Barcelona Clinic Liver Cancer; *MELD* model of end-stage liver disease; *ECOG * Eastern Cooperative Oncology Group; *CPT  *Child–Pugh

### Predictors of progression free survival

Kaplan–Meier survival analysis (Supplementary file 1) revealed either diarrhoea or hypertension as an AE was associated with improved PFS. Patients with diarrhoea had a median PFS of 6.2 months versus 5.6 in those without diarrhoea (*p* = 0.04). In patients who developed new or worsening hypertension the median PFS was 8.2 months versus a median PFS of 5.5 months in patients who did not develop hypertension (*p* = 0.01). Additionally, the requirement for a dose reduction was also associated with improved PFS (8.2 vs 4.1 months respectively (*p* < 0.01)).

### Regression analysis

Predictors of improved PFS on univariate analysis were dose reduction (HR 0.49, 95% CI 0.33–0.73, *p* < 0.01), withholding treatment (HR 0.56, 95% CI 0.34–0.94, *p* = 0.02), older age (HR 0.98, 95% CI 0.96–0.99, *p* = 0.02), increased baseline albumin level (HR 0.95, 95% CI 0.92–0.98, *p* < 0.01), and AEs of either diarrhoea (HR 0.60, 95% 0.40–0.91, *p* = 0.01) or hypertension (HR 0.83, 95% 0.70–0.99, *p* = 0.04). A higher baseline CP score was associated with a shorter PFS (HR 1.27, 95% CI 1.02–1.58, *p* = 0.03).

On multivariate analysis, dose reduction (HR 0.45, 95% CI 0.29–0.68, *p* < 0.01), older age (HR 0.96, 95% CI 0.94–0.98, *p* < 0.01) and a higher baseline CP score (HR 1.24, 95% CI 1.01–1.52, *p* = 0.04) remained as independent and significant predictors of PFS (Table 4).

**Table 4:** Univariate and multivariate analysis of factors predictive of disease progression

	Univariate analysis			Multivariate analysis		
	HR	95% CI	*p*	aHR	95% CI	*p*
Dose reduction (yes vs no)	0.49	0.33–0.73	< 0.01	0.45	0.29–0.68	< 0.01
Age (per year increase)	0.98	0.96–0.99	0.02	0.96	0.94–0.98	< 0.01
Baseline CP score (per one-point increase)	1.27	1.02–1.58	0.03	1.24	1.01–1.52	0.04
Treatment withheld (yes vs no)	0.56	0.34–0.94	0.02			NS
Diarrhoea as an adverse event (yes vs no)	0.60	0.40–0.91	0.01			NS
HTN as an adverse event (yes vs no)	0.83	0.70–0.99	0.04			NS
Baseline albumin (per g/L increase)	0.95	0.92–0.98	< 0.01			NS

*BCLC* Barcelona Clinic Liver Cancer; *CPT *Child–Pugh

### Adverse events

During treatment, 130 patients experienced an AE of any grade (83.9%), of which 48 (28.4%) were grade three or four. Fifty-eight patients required permanent cessation of therapy due to an AE (37.4%). The most common AE was new or worsening hypertension experienced by 55 patients (35.5%), with 43 patients (27.7%) requiring anti-hypertensive medication. The percentage of patients with pre-existing hypertension before commencing lenvatinib was 49%. Sixteen patients (10.3%) required thyroid replacement therapy due to hypothyroidism. The remaining AE, their frequencies and grades are summarised in Table 5. Three patients (1.9%) experienced a grade four AE and these were malignant hypertension, duodenal perforation and HCC necrosis with heart failure. There was one sudden unexplained death while on therapy (day 17 of therapy).

**Table 5:** Frequency and grade of adverse events post commencement of Lenvatinib

Adverse event	Overall (%)	Grade 1 (%)	Grade 2 (%)	Grade 3 (%)	Grade 4 (%)
Any adverse event	130 (83.9)	95 (61.3)	77 (49.7)	46 (29.7)	3 (1.9)
Hypertension	55 (35.5)	14 (9.0)	18 (11.6)	22 (14.2)	1 (0.6)
Hypothyroidism	23 (14.8)	7 (30.4)	15 (65.2)	1 (4.3)	0 (0)
Diarrhoea	38 (24.5)	24 (63.2)	14 (36.8)	0 (0)	0 (0)
Hand-food syndrome	33 (21.3)	17 (51.5)	15 (45.5)	1 (3.0)	0 (0)
Dysphonia	13 (8.4)	9 (69.2)	4 (30.8)	0 (0)	0 (0)
Anorexia	40 (25.8)	18 (45.0)	14 (35.0)	8 (20.0)	0 (0)
Fatigue	52 (33.5)	15 (28.8)	22 (42.3)	15 (28.8)	0 (0)
Nausea/vomiting	27 (17.4)	14 (51.9)	10 (37.0)	3 (11.1)	0 (0)
Weight loss	15 (9.7)	9 (60.0)	6 (40.0)	0 (0)	0 (0)
Rash	7 (4.5)	5 (71.4)	2 (28.6)	0 (0)	0 (0)
Mouth ulcers	3 (1.9)	1 (33.3)	1 (33.3)	1 (33.3)	0 (0)
Hepatic decompensation	7 (4.5)	0 (0)	5 (71.4)	2 (28.6)	0 (0)
Myalgia	4 (2.6)	4 (100.0)	0 (0)	0 (0)	0 (0)
Other	9 (15.5)	9 (37.5)	5 (20.8)	7 (29.2)	2 (8.3)*

Grade 4 adverse events in other category included duodenal perforation and HCC necrosis with heart failure

## Discussion

This retrospective multi-centre study is the first to examine real-world characteristics and outcomes of lenvatinib use for advanced HCC patients in Australia. This study population is among the largest published real-world lenvatinib treated cohorts globally and includes a relatively long follow-up period for analysis of predictors of OS and PFS. Patients were drawn from eleven Australian tertiary referral centres and represent a heterogenous multi-ethnic population representative of the Australian community. There was a variety of etiologies of liver disease with higher rates of NAFLD (25%) and HCV infection (40%) than seen in the registration trial of lenvatinib where 50% of subjects had HBV infection [8].

Median OS and PFS in our cohort were 7.7 months (IQR: 4.8–11.6) and 5.3 months (IQR: 2.8–9.2) respectively, which were lower than those seen in the REFLECT trial (13.6 months and 7.4 months) [8]. This likely relates to the inclusion of patients outside trial criteria, specifically those with main portal vein invasion (*n* = 44, 24.8%) or CP B or C disease (*n* = 29, 18.7%). A total of 27 patients (17.4%) had prior exposure to sorafenib. This group did not have worse overall survival in this analysis. The time point analysed in our cohort may also reflect a period where there was a relative lack of other therapeutic options for patients with advanced HCC in Australia which may have also contributed to more treatment experienced patients. The proportion of patients with CP B or C cirrhosis and prior sorafenib exposure in our cohort were similar to the ranges seen in other real-world studies; 9.0–39.1% and 25.0–39.5%, respectively [12–18]. Reassuringly the subgroup of patients in our cohort with CP A disease had a median OS of 12.5 months, which was comparable to that seen with in the lenvatinib arm of the REFLECT trial (13.6 months) [8]. Indeed, a German cohort has demonstrated that patients receiving lenvatinib who meet REFLECT inclusion criteria have greater OS than those who do not, suggesting caution is warranted in this population with decompensated liver disease [22].

Lenvatinib related AEs were common with approximately 85% of patients experiencing a treatment related AE, but only 30% and 2% of patients experienced a grade 3 or 4 AE respectively, confirming that lenvatinib remains a safe and well-tolerated therapeutic option. Our AE rate is numerically lower than the REFLECT trial where 99% of patients experienced a treatment emergent AE and 75% a grade 3 or 4 AE, however this may be due to the retrospective nature of our study limiting the ability to record all AEs. Our AE rates are similar to other real-world studies from Korea and Japan which reported relatively few grade 3 or 4 AEs [8, 12–16].

Following several years of sorafenib use, data emerged demonstrating that development of skin toxicity, hand–foot syndrome (HFS) or diarrhoea predicted better outcomes [23, 24]. Our results demonstrate a similar finding for lenvatinib with patients who developed hypertension or diarrhoea surviving almost two times longer than those that did not (16.2 vs 9.4 months (*p* = 0.02) and 17.5 vs 10.1 months (*p* = 0.08) respectively). Additionally, patients who needed a dose reduction related to an AE of any cause had survival approximately 2.5 times longer compared to those who did not (19.6 vs 7.8 months (*p* < 0.01) respectively). Based on these results, it may be appropriate to dose titrate to patient tolerance as a reduction in dose does not portend worse outcomes. These findings are supported by other studies. In a Korean cohort (*n* = 111) [25], diarrhoea and HFS were associated with higher PFS but did not correlate with survival, while a Japanese cohort (*n* = 52) [26] demonstrated that the occurrence of hypothyroidism predicted improved survival. Post hoc analysis of the REFLECT study which is yet to be published in full also appears to support this finding [27]. Therefore, the development of AEs may be a marker for therapeutic compliance and in vivo drug activity, resulting in increased PFS and OS.

Although the role of lenvatinib in the treatment of HCC is changing with the introduction of immunotherapy, the medication continues to play a role in patients who may be inappropriate for immunotherapy. Indeed, in Australia where both agents are available and funded, lenvatinib still represents approximately 25% of government subsidised prescriptions for patients with HCC [9]. In some countries where immunotherapy is not subsided, lenvatinib remains the standard of care. The results of LEAP002, a phase 3 randomised controlled trial comparing lenvatinib plus pembrolizumab versus lenvatinib and placebo are awaited [28] to inform the efficacy of expanded use of lenvatinib.

Our study has a number of strengths. The multicentre nature of the study allowed for one of the largest retrospective studies of lenvatinib to date with a heterogenous population. The relatively long follow-up time also allowed for further analysis of factors associated with OS and PFS to help guide further practice.

A limitation of our study is its retrospective nature which relies on the accuracy and completeness of data found in medical records. However, retrospective data collection provided the opportunity to maximise our study cohort and follow-up time. Furthermore, a hard end-point, OS, was chosen as the primary study outcome to minimise subjectivity.

Our study population had a higher prevalence of males with HCC than females; higher than the reported gender disparity in other studies. Although gender did not impact OS or PFS in our statistical analysis, further studies are required to confirm if gender impacts outcomes in advanced HCC [29, 30]. Lastly, the non-controlled nature of the study may have led to additional support and care offered to patients who developed AEs (hypertension or diarrhoea), however this is unlikely to account for the differences in reported outcomes.

## Conclusion

In real-world practice in Australia, lenvatinib was prescribed outside of the reimbursed indication in up to 20% of patients. The median overall survival of 12.5 months in patients with well compensated liver disease was more than twice that found in patients with CP B/C cirrhosis. Lenvatinib was safe and well-tolerated in our cohort. Development of hypertension and diarrhoea, and the subsequent need for dose reduction were independently associated with improved overall survival.

## Supplementary Information

Below is the link to the electronic supplementary material.

Supplementary file1 (DOCX 37 KB)

## Abbreviations

HCC: Hepatocellular carcinoma
BCLC: Barcelona clinic liver cancer
OS: Overall survival
PFS: Progression free survival
IQR: Interquartile range
HR: Hazard ratio
95% CI: 95% Confidence intervals
CP: Child–Pugh
MELD: Model of end-stage liver disease
HTN: Hypertension
AE: Adverse event
HFS: Hand food syndrome
