# Supplementary material for: Lenvatinib for the treatment of hepatocellular carcinoma—a real-world multicenter Australian cohort study
Source: Hepatol Int. 2022 Aug 25;16(5):1170–8. doi: 10.1007/s12072-022-10398-5 (PMC9525325; doi:10.1007/s12072-022-10398-5)
Supplement: Supplementary file 1 — Supplementary file1 (DOCX 37 KB) [file 12072_2022_10398_MOESM1_ESM.docx]

# **Supplementary Material**

***Supplementary figure 1*** *-* Kaplan Meier *curves* for progression free survival stratified by treatment emergent diarrhoea (a) new or worsening hypertension (b) and the need for a dose reduction due to AE (c)
